# Earlier Development of Analytical than Holistic Object Recognition in Adolescence

**DOI:** 10.1371/journal.pone.0061041

**Published:** 2013-04-05

**Authors:** Elley Wakui, Martin Jüttner, Dean Petters, Surinder Kaur, John E. Hummel, Jules Davidoff

**Affiliations:** 1 Department of Psychology, Goldsmiths, University of London, London, United Kingdom; 2 School of Psychology, University of East London, London, United Kingdom; 3 Psychology, School of Life and Health Sciences, Aston University, Birmingham, United Kingdom; 4 Department of Psychology, University of Illinois, Urbana, United States of America; University of Tokyo, Japan

## Abstract

**Background:**

Previous research has shown that object recognition may develop well into late childhood and adolescence. The present study extends that research and reveals novel differences in holistic and analytic recognition performance in 7–12 year olds compared to that seen in adults. We interpret our data within a hybrid model of object recognition that proposes two parallel routes for recognition (analytic vs. holistic) modulated by attention.

**Methodology/Principal Findings:**

Using a repetition-priming paradigm, we found in Experiment 1 that children showed no holistic priming, but only analytic priming. Given that holistic priming might be thought to be more ‘primitive’, we confirmed in Experiment 2 that our surprising finding was not because children’s analytic recognition was merely a result of name repetition.

**Conclusions/Significance:**

Our results suggest a developmental primacy of analytic object recognition. By contrast, holistic object recognition skills appear to emerge with a much more protracted trajectory extending into late adolescence.

## Introduction

The present paper is concerned with the development of the long-term representation of visual shapes used for the recognition and naming of common objects. Despite indications of remarkably selective visual representations even in 3-to 4-month-olds (e.g., [1], [2]) there is increasing evidence that object recognition may only become fully adult-like late into childhood and adolescence ([3], [4], [5]; for a review see [6]). However, the cause of this protracted development is not well understood. In the past, similar claims have been made more frequently for face recognition skills where there is substantial evidence for a continuing improvement deep into the second decade of life (e.g., [7], [8], [9]). Much of the late developing skills for face recognition has been attributed to the processing of spatial relations; indeed, similar protracted configural skills also affect the recognition of non-face objects in children up to an age of 12 years [5].

Here we present another aspect of object recognition that has a tardy development and one that is at first a surprise. Popular image-based approaches to object recognition (e.g., [10], [11], [12]) propose ‘holistic’ representations that are ‘all-in-one’, or view-like, where object features are represented in terms of their literal position in a pictorial, two-dimensional coordinate system. Indeed, all object recognition can be explained from such holistic representations [13] though they are alternatively contrasted with the classical idea of an analytical, structural object description based on certain volumetric primitives and their categorical relations [14], [15]. One point to note is that the use of the term holistic in studies of object recognition differs from the one prevailing in research into face recognition. For the latter, ‘holistic’ typically implies a more sophisticated form of configural processing in which the recognition of an individual facial feature interferes with the context provided by other, neighbouring features (for a review see e.g. [9]). Depending on the task the interference with facial context may either facilitate recognition of the target feature (e.g., [16]) or impede it (e.g., [17]). Such configural-holistic processing for faces matures early and reaches adult level by the age of six [18], [19]. Whether its presence implies a specific representational format for faces that differs from that for non-face objects has been controversial (see e.g. [20] but [21]) and remains an issue for further research. However, with respect to holistic representations as used in object recognition, such simple representations too would be presumed to be acquired early in cognitive development.

To differentiate the development of holistic and analytic object recognition, we make use of Hummel’s [22] dual route model of object recognition (cf. also [23]). According to the model, objects are represented and processed in two different formats - analytical and holistic – that are combined into a hybrid representation in long-term memory. The analytic pathway involves explicit structural descriptions based on an object’s parts and their relations whereas the holistic pathway is view-like. An important characteristic of Hummel’s model are the different attentional demands of the two processing routes. The analytical route is assumed to require visual attention to mediate the dynamic binding between the segmented parts of an object and the relations that hold between them, thus providing a structural object description that is invariant to transformations like left-right reflection and some rotations in depth. By contrast, the holistic pathway is considered to operate independently of attention as the object’s local shape features are statically bound by their relative location within a so-called surface map. The surface map preserves topological relations of these features, resulting in a holistic representation that is sensitive to left-right reflection and rotation.

Predictions of the dual-route model concerning the modulating effect of attention have been tested in repetition-priming experiments. Such studies typically compare the effect of two briefly presented prime stimuli: one attended and the other ignored. Priming is assessed in terms of the facilitation for naming a subsequently presented probe stimulus. According to the model, holistic priming could in principle be observed both for the attended and the ignored prime stimulus. However, given the view-like object representation used by the holistic route the priming would critically depend on the pictorial identity of prime and probe. By contrast, analytic priming should result only from the attended prime stimulus. Due to the more abstract object format implied by the analytic route, this priming should tolerate image differences between probe and prime as long as those permit at least a partial matching of the underlying structural representations. In line with these predictions, analytic - but not holistic - priming has been found to be robust to a variety of manipulations of object view including left-right mirror reflection [24], image splitting [25], plane rotation [26] and rotation in depth [27]. Moreover, the analytic priming in these studies strictly depended on attentional allocation and was absent for ignored primes, whereas holistic priming was observed for both attended and ignored primes as suggested by the dual-route model. Further, neural evidence for the model comes from the results of a recent study assessing repetition priming in conjunction with fMRI [28]. These suggest a mapping of the two routes on the ventral and dorsal visual-processing streams [29], with ventral stream regions being implied in both analytical and holistic processing and holistic processing only in dorsal stream regions.

The extent to which the holistic and analytic routes contribute to object recognition in the developing mind remains uncertain. With regard to the analytic route there is good evidence that even young children and toddlers give special importance to part information for object categorization and naming (e.g., [30], [31], [32], [33], [34]), and that 7- to 8-year-olds perform already close to adult levels when recognizing part changes in familiar objects [5]. However, no previous research has considered the developmental trajectory of the holistic processing route. As this route operates automatically and irrespective of attentional allocation it has been proposed (e.g., [22]) to account for the surprising speed of object recognition well documented at behavioural [35] and neurophysiological [36] levels. On that account, the fast holistic route could be seen as a more elementary and ontologically primary path compared to the slower, more elaborate and more abstract processing provided by the analytical pathway. Nevertheless, Thoma and Davidoff [37] demonstrated that holistic priming is not observed with unfamiliar object views as prime stimuli. Holistic priming effects therefore appear to depend on long-term memory representations of visual shape and to differ from the facilitation observed in sequential visual matching. The latter would be expected to provide an advantage also in case of unfamiliar stimuli, i.e., in the absence of any long-term object representation (e.g., [38], [39], [40]). Thus, holistic processing may require prolonged visual experience and, as a consequence, show a late maturation. In our experiments, we tested that hypothesis employing a repetition-priming paradigm which we adapted for use with children in our target age range from 7 to 11 years. The children’s performance was contrasted with that of young adults as control and reference.

In Experiment 1, we assessed the role of attention in repetition priming for identical and left-right reflected images. According to the dual route model, analytical and holistic processing should make independent contributions to priming. More specifically, analytical processing is demonstrated by attended but not ignored images priming themselves and their left- right reflections to the same extent. Holistic processing should manifest itself as an additional advantage of identical over left-right reflected images and should be equal for attended and ignored primes. We used these behavioural markers to contrast the involvement of analytical and holistic processing in children and adults, and to establish the relative dominance of the two routes during the development of object recognition.

## Experiment 1

### Methods

#### Ethics statement

All experimental procedures were approved by the Ethics Committees of Goldsmiths College and Aston University prior to the commencement of the study. Informed consent was obtained from all participants, in case of children also from the parents or guardians and the participating schools.

#### Participants

There were three age groups: Twenty-eight 7/8-year-olds (mean age 7 years 9 months; 13 females, 15 males), twenty-eight 11/12-year-olds (mean age 11 years 9 months; 12 females, 16 males), and twenty-eight adult volunteers (mean age 22 years 9 months; 20 females, 8 males). The children were drawn from state schools in Birmingham and London, UK. The adults were recruited among undergraduate Psychology students at Goldsmiths College. They received course credit for participation.

#### Stimuli and apparatus

We used a set of 56 black-and-white line drawings of asymmetrical familiar animals and non-face objects. The stimuli were taken from the Rossion and Pourtois [41] version of the Snodgrass and Vanderwart [42] picture set.

They were presented on a 15.6 inch monitor with a screen size (height×width) of 20.4×33 cm, subtending a visual angle of 19.5°×31.5° at the viewing distance of 60 cm. The images were standardized in size to subtend 4.5°×4.5°. For each object, a mirror-reflected version was created using Adobe Photoshop. Voice responses were detected through a dynamic trigger microphone attached to an interface box. Stimulus presentation and response collection were controlled by an Eprime 1.1 (Psychology Software Tools, Inc.) script running on a Toshiba laptop computer.

#### Procedure

**Figure 1:**
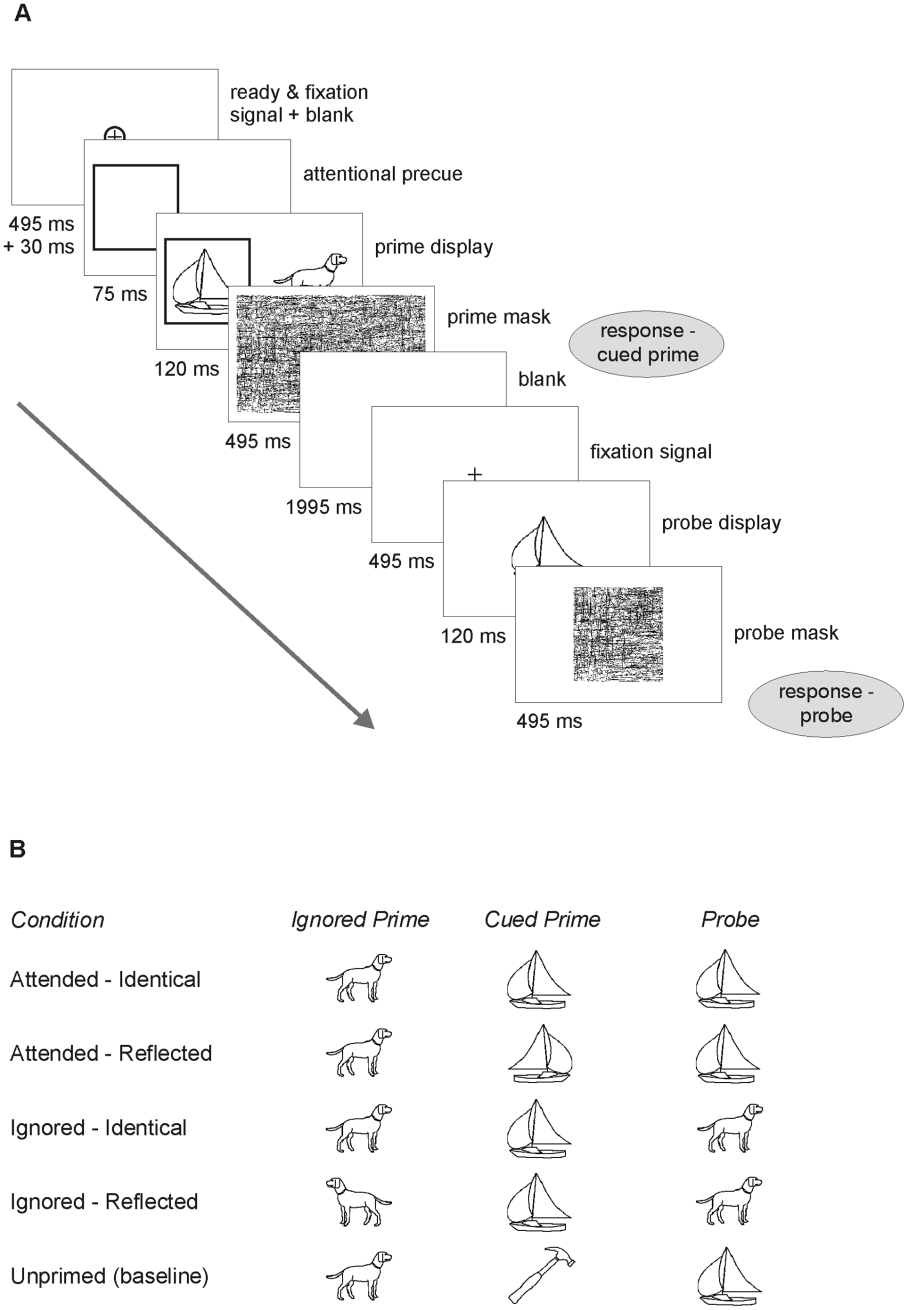
Sequence of events and stimulus conditions in Experiment 1. As indicated in (A) in each trial the participant had to name the objects shown as cued prime and as probe. The five conditions concerning the relation between probe and prime display are illustrated in (B). Accordingly, the probe could be identical to either the cued (attended) prime, the ignored (unattended) prime, or to their respective mirror-reflected versions. For each condition, priming was measured in terms of the response time reduction relative to the unprimed baseline condition.

Participants were tested using the repetition priming paradigm of Stankiewicz et al. [24] (cf. Fig. 1a). Each trial sequence began with a centrally presented circle (size: 0.032°) as a ready signal. In a change to Stankiewicz et al.’s original procedure, and to make the procedure feasible for the use with children, the experimenter rather than the participant initiated the rest of the trial. First, a central fixation cross appeared for 495 ms followed by a blank screen for 30 ms. Next an attentional precue, a square (4.5°×4.5°), was shown centred either 4° to the left or to the right from fixation, counterbalanced across trials for each participant. After 75 ms two prime images were simultaneously displayed for 120 ms, with the to-be-attended image inside the square and the to-be-ignored image at an equal distance to the other side of the screen. They were followed by a random-line mask (15.6°×15.6°) for 495 ms. The entire prime display lasted less than 200 ms, a duration too short to permit a voluntary saccade to the cue or any of the two prime images. Participants were required to name the cued (attended) object as quickly and accurately as possible.

After the prime display, a blank screen was presented for 1995 ms. The probe sequence started with a fixation cross, shown for 495 ms and a blank screen shown for 30 ms. The probe image was then displayed in the centre of the screen for 150 ms followed by a random-line mask (4.6°×4.6°) for 495 ms. Participants were required to name the probe object. Feedback on the names of the prime and the probe was displayed and the experimenter coded the accuracy of the responses using the buttons of a computer mouse; this also initiated the next trial.

The experimental session consisted of a series of 12 practice trials and 24 experimental trials. The practice trials served to familiarize the participant with the task and the procedure. To children, the experiment was introduced as a computer game to ensure their interest and motivation. In each trial there were five possible stimulus conditions concerning the relation between prime and probe stimuli (Fig. 1b): The probe could be identical to the attended prime (condition: Attended–Identical) or its mirror reflection (Attended-Reflected); or it could be identical to the unattended prime (Ignored-Identical) or its mirror reflection (Ignored-Reflected), or it could depict a completely different object (Unprimed). Each picture was used only once in each experimental session, either as a prime or a probe. Across observers, all stimuli appeared equally often in all stimulus conditions. The practice trials involved a set of images different from the experimental set.

### Results and Discussion

Performance was analyzed in terms of the extent of priming, which was calculated as the participant’s mean response time at the probe in the unprimed (baseline) condition minus the mean response time in the corresponding experimental condition. Trials on which there were voice key errors or either the prime or probe response was incorrect (17.3%) were excluded from the analysis. Only responses within ±3 MAD from the median latency were included in the analysis.

**Figure 2:**
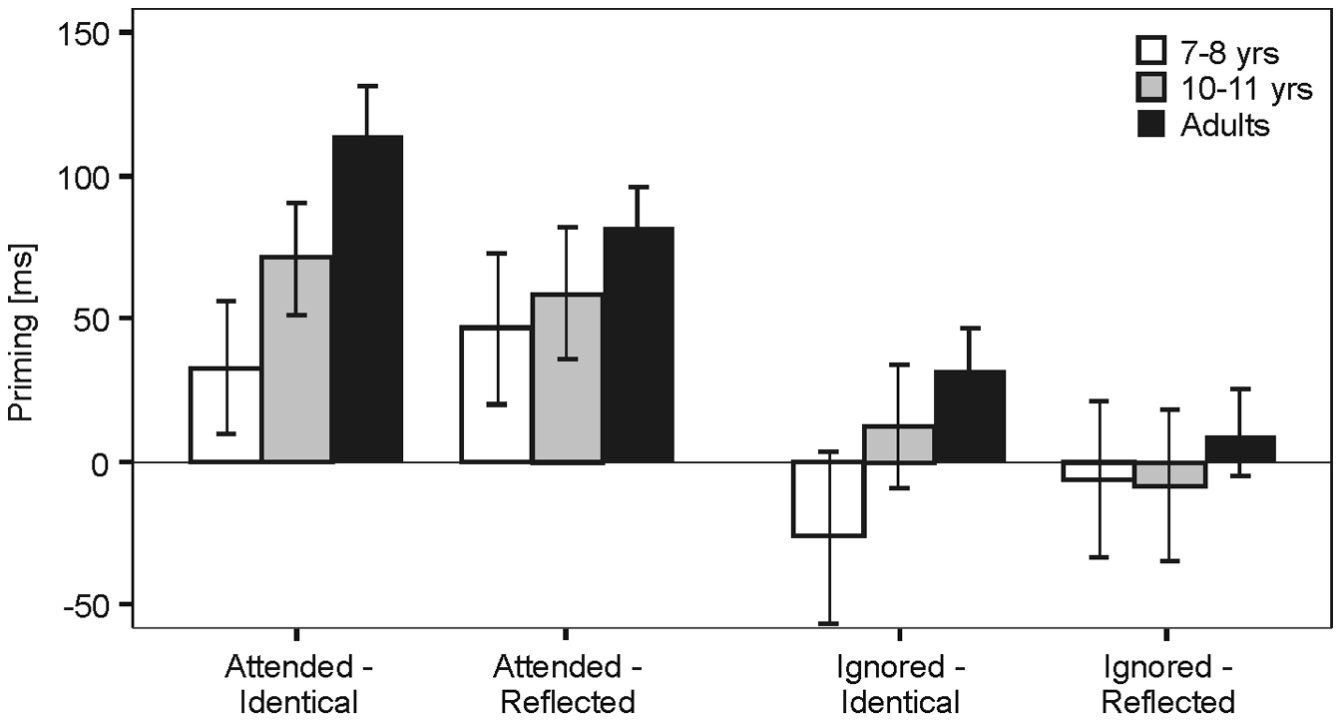
Results for Experiment 1. Priming means as a function of attentional allocation (Attended vs Ignored) and view (Identical vs Reflected) for each age group in Experiment 1. Error bars represent standard errors.

Figure 2 shows the mean priming for each age group as a function of attentional allocation and probe view. The priming data were entered into a 2 (Attention: Attended vs. Ignored)×2 (View: Identical vs. Reflected)×3 (Age: Adults vs. 11–12 years vs. 7–8 years) mixed ANOVA with Age as between factor. The analysis gave a significant main effect of Attention [F(1,79) = 28.86, p<.001, η_p_^2^ = .268] and a significant interaction between View and Age [F(2,79) = 3.53, p<.05, η_p_^2^ = .082]. Follow-up t-tests showed that only adults showed an effect of View with priming from identical images greater than from reflected images [72.9 ms vs. 45.5 ms; t(27) = 3.15, p<.005]. All other main effects were non-significant [Age: F(2,79) = 1.18, p = .17; View: F(1,79) = .89, p = 0.35], as were all remaining interactions (ps >.67).

The age dependent effects on priming could not be explained by differences in the number of correct naming trials included in the analysis which might have affected the statistical power to detect priming in younger children. An additional one-way ANOVA of the relative proportion of such correct trials (group means: 7–8 years:.82; 11–12 years:.83; adults:.87) across the three age groups proved non-significant (F(2,81) = 1.91; p = .16).

The results demonstrate a clear dissociation between children and adults regarding the impact of priming on object recognition. While all age groups showed a reliable facilitation of naming for attended prime stimuli, it was only adults who showed an additional benefit from identical views of probe and prime, regardless of attentional allocation. The additional facilitation for adults comes from the benefit of having automatically activated holistic representations; this advantage is absent in the two groups of children even for stimuli to which they are directing their attention. The contrasting results cannot be explained in terms of the well-documented limitations in children’s ability to voluntarily deploy attention to specified regions in the visual field (e.g., [43], [44]). When given a locational precue - as in the present study - even 5-year olds have been shown to use attention to filter out distractors as well as adults [44]. Indeed, the consistent facilitation observed for attended primes in both the 7 to 8 and 10 to 11-year-olds demonstrates the effectiveness of that attentional manipulation.

The observed pattern of results also cannot be accounted for by assuming that the prime display constitutes a higher perceptual load in children than in adults, thus potentially leading to a reduced accessibility of the unattended, to-be-ignored prime stimulus (e.g., [45]). Such an explanation fails to explain the interaction between View and Age, in particular the notable absence of an additional facilitation of attended identical relative to attended mirror-reflected primes in both groups of children (ps >0.40) - a result that contrasts with the significant facilitation found for adults [t(27) = 2.38, p<0.05].

The observed pattern of priming effects in children, i.e., the lack of priming by unattended identical primes and the equivalence of priming for attended identical and mirror-reflected primes, suggests that children – unlike adults – do not employ view-like, holistic representations for object naming. Rather they only draw on representations that are invariant to certain structural manipulations like mirror reflection. In principle, such representations would be mediated by analytical structural descriptions. However, our data could also simply arise as a consequence of name repetition. To test this possibility, we conducted a second experiment.

## Experiment 2

In Experiment 1, children only showed priming to attended stimuli. The lack of a difference between the Identical and the Reflected condition could indicate a mediating visual object representation that is sufficiently abstract to afford invariance to reflection. Alternatively, it could be the consequence of name priming, i.e., induced by the fact that the prime and probe referred to the same concept regardless of any visual transformation. For adults, previous research has shown that priming from attended images is largely visual in nature (e.g., [46], [24], [25]) and therefore not the result of name repetition. However, given the striking differences in the priming pattern observed for children in Experiment 1 an additional study was run to directly compare the impact of visual priming and name priming.

In Experiment 2, the Identical view condition of Experiment 1 was replaced with a Same-Name-Different-Examplar (SNDE) condition in which prime and probe image showed objects with the same basic-level category name (cf. [47]) but different shapes. For example, a prime image showing an upright piano would be followed by probe image showing a grand piano, thus eliciting the same response (“piano”) for a stimulus with a different shape.

The images of the Reflected condition were the same as in Experiment 1, as were the attentional manipulations. Thus, Experiment 2 provided a direct comparison of the effects of name priming (as assessed in the SNDE condition) against analytical visual priming (as assessed in the Reflected condition). If any of the priming observed for attended, mirror-reflected objects in Experiment 1 was specifically visual, Experiment 2 should yield significantly less priming for attended stimuli in the SNDE relative to the Reflected condition. Conversely, if that priming was the result of name repetition then attended SNDE primes should be at least as effective as attended mirror-reflected primes.

### Methods

#### Ethics statement

All experimental procedures were approved by the Ethics Committees of Goldsmiths College and Aston University prior to the commencement of the study. Informed consent was obtained from all participants, in case of children also from the parents or guardians and the participating schools.

#### Participants

Three age groups took part, each consisting of 28 participants: 7/8-year-olds (mean age 8 years 0 months; 12 females, 16 males), 10/11-year-olds (mean age 10 years 5 months; 17 females, 11 males), and adult volunteers (mean age 27 years 9 months; 18 females, 10 males). The children were drawn from state schools in Birmingham and London, UK. The adults were recruited among undergraduate Psychology students at Goldsmiths College. They received course credit for participation.

#### Stimuli and apparatus

A set of 112 black-and-white line drawings of asymmetrical familiar animals and objects was used. Stimuli were divided into 56 pairs such that each object had a SNDE counterpart, i.e., an exemplar of a different shape but with the same name, i.e., the same basic-level category name. Half of them were identical to the stimuli used in Experiment 1. The SNDE exemplars were line drawings of a similar style, and were adopted from Thoma et al. [25] (Experiment 2).

#### Procedure

The procedure used in Experiment 2 was identical to that employed in Experiment 1, with the exception that identical primes were replaced by SNDE primes, both in the attended and non-attended condition. Across participants, all objects appeared equally often in each prime-probe condition.

### Results and Discussion

Performance was again analysed in terms of the observed priming. As in Experiment 1, trials on which there were voice key errors or either the prime or probe response was incorrect (13.7%) were excluded from the analysis. As in Experiment 1, only responses ±3 MAD from the median were included in the analysis. Following this criterion, two participants in the age group 7–8 years were classified as outliers and removed from the data prior to the statistical analysis.

**Figure 3:**
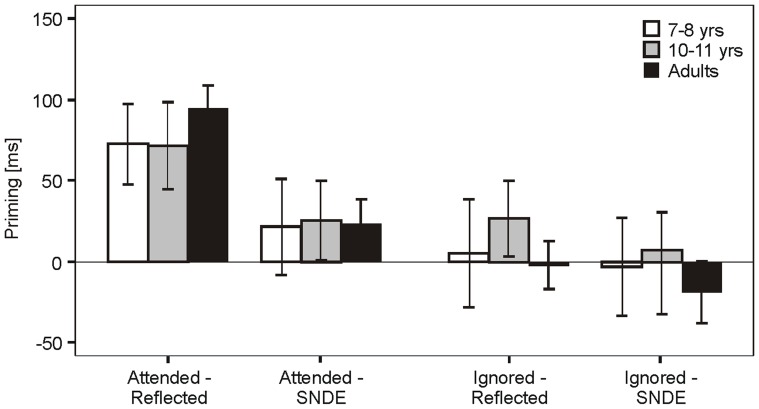
Results for Experiment 2. Priming means as a function of attentional allocation (Attended vs Ignored) and view (Same-Name-Different-Exemplar vs Reflected) for each age group in Experiment 2. Error bars represent standard errors.

The mean priming in Experiment 2 for each age group as a function of attentional allocation and probe view is shown in Figure 3. The priming data were entered into a 2 (Attention: Attended vs. Ignored)×2 (View: Reflected vs. SNDE)×3 (Age: Adults vs. 11–12 years vs. 7–8 years) mixed ANOVA, again with Age as between factor. It gave significant main effects of Attention [F(1,76) = 13.09, p<.001, η_p_^2^ = .147] and View [F(1,76) = 6.42, p<.01, η_p_^2^ = .078] but not of Age [F(2,76) = .34, p = .71]. In addition, there was a strong trend in the interaction between Attention and View [F(2,76) = 3.5, p = .06, η_p_^2^ = .044]. To follow up the interaction the data were collapsed across age groups. Paired t-tests showed that priming in the attended reflected condition (73.7 ms) was significantly greater than in the attended SNDE condition [24.0 ms; t(27) = 4.08, p<.001]. All other interactions did not reach statistical significance p>.6. By contrast, there was no priming for ignored stimuli. Priming by ignored reflected stimuli and ignored SNDE stimuli were both not significantly different from zero (ps >0.5; one-sample t-test).

Experiment 2 showed that a probe object was primed more by an attended mirror-reflected version of the same image than by a different exemplar of the same basic-level category. In both cases, participants responded with the same name in probe and prime trials, which suggests that the advantage of the mirror-reflected condition was the result of visual rather than concept priming. These findings did not depend on age group and are consistent with previous work involving adult observers only (e.g., [46], [24], [25]). They support the conclusion that the priming in the attended condition observed for children in Experiment 1 was mediated by visual representations, which within the context of the dual route model can be qualified as being analytic in the sense that they are invariant to certain structural manipulations like mirror reflection.

## General Discussion

The present investigation is the first developmental study to directly compare analytical and holistic processing in object recognition. With respect to the less cognitive task of perceptual organization, substantial previous research has explored the role of age. In particular, the ability to integrate local visual features spatially across the visual field (e.g., [48], [49]) and the sensitivity to global structure in hierarchical visual stimuli (e.g., Navon patterns) has been found to continue to develop into late childhood and early adolescence (e.g., [50], [51]). Thus, the developmental evidence suggests that the precedence for global shape in visual percepts typically observed in adults may only develop late ontogenetically. Our experiments go beyond that research by demonstrating a precedence of analytic processing for object recognition proper, i.e., the matching of a visual percept to a memory representation. Across our target age range of 7–11 years, the participating children showed consistent significant effects of analytical priming suggesting the availability of structural, part-based object representations. However, more dramatically, the children equally consistently failed to show any evidence of holistic priming, thus indicating a much reduced impact of holistic representations on object recognition compared to those of the analytic route. Holistic object recognition skills appear to emerge with a protracted trajectory that extends into late adolescence.

Why is the analytical path the primary route used by children? Unlike for the identification of faces, the recognition of objects typically occurs at the so-called basic level [47], where parts and their spatial configuration provide reliable information for successful categorization and naming. The analytic object processing mediating recognition at this level has been proposed to involve highly regularized structural descriptions [14], [22]. While the formation of such descriptions requires attentional binding, the resulting representations are robust against many accidental changes in object view (for example, due to a small rotation in depth) thus facilitating their consolidation in long-term memory. Moreover, such descriptions offer – even with relatively few parts - substantial representational power for basic-level categories [14] which may make them a preferred route for knowledge extension in children.

A fundamental implication of our data is that holistic representations are not those primarily used in object recognition. Holistic object representations have been conceptualized as collection of features bound together implicitly through their coordinates in a quasi-pictorial, view-like format (e.g., [10], [11], [12]). While such representations are structurally simpler and appear less dependent on directed visual attention, their consolidation in long-term memory may be hindered by their limited invariance to manipulations of object view. Current view-based models do not directly address issues of development but object learning experiments in adult observers suggest that the acquisition of view-based object representations is predominantly driven by statistical learning (e.g., [11]). During such learning, distinct views emerge as a result of gradual familiarization with clusters of viewpoint-specific features. Thus, holistic object recognition skills may crucially depend on repeated exposure to the same stimulus presented from the same viewpoint which would explain the need of a prolonged period of consolidation. In line with this notion, repetition priming experiments have found holistic priming only for familiar probe views but not unfamiliar ones [37].

While holistic object recognition skills take surprisingly long to develop, once acquired they may offer a shortcut to enable particularly fast object recognition [35], [36]. They may also provide – through possible involvement of the dorsal pathway [28] - a facilitation of hand movements that subserve the interaction with objects [29] even though the neurological basis underlying their development is still awaiting elucidation.
